# Frequency format diagram and probability chart for breast cancer risk communication: a prospective, randomized trial

**DOI:** 10.1186/1472-6874-8-18

**Published:** 2008-10-20

**Authors:** Karthik Ghosh, Brianna J Crawford, Sandhya Pruthi, Constance I Williams, Lonzetta Neal, Nicole P Sandhu, Ruth E Johnson, Dietlind Wahner-Roedler, Marcia K Britain, Stephen S Cha, Amit K Ghosh

**Affiliations:** 1 Breast Diagnostic Clinic, Mayo Clinic, Rochester, Minnesota, USA; 2 Division of Biostatistics, Mayo Clinic, Rochester, Minnesota, USA; 3 Division of General Internal Medicine, Mayo Clinic, Rochester, Minnesota, USA

## Abstract

**Background:**

Breast cancer risk education enables women make informed decisions regarding their options for screening and risk reduction. We aimed to determine whether patient education regarding breast cancer risk using a bar graph, with or without a frequency format diagram, improved the accuracy of risk perception.

**Methods:**

We conducted a prospective, randomized trial among women at increased risk for breast cancer. The main outcome measurement was patients' estimation of their breast cancer risk before and after education with a bar graph (BG group) or bar graph plus a frequency format diagram (BG+FF group), which was assessed by previsit and postvisit questionnaires.

**Results:**

Of 150 women in the study, 74 were assigned to the BG group and 76 to the BG+FF group. Overall, 72% of women overestimated their risk of breast cancer. The improvement in accuracy of risk perception from the previsit to the postvisit questionnaire (BG group, 19% to 61%; BG+FF group, 13% to 67%) was not significantly different between the 2 groups (*P *= .10). Among women who inaccurately perceived very high risk (≥ 50% risk), inaccurate risk perception decreased significantly in the BG+FF group (22% to 3%) compared with the BG group (28% to 19%) (*P *= .004).

**Conclusion:**

Breast cancer risk communication using a bar graph plus a frequency format diagram can improve the short-term accuracy of risk perception among women perceiving inaccurately high risk.

## Background

A patient's knowledge of risks and benefits is crucial to informed decision making [1]. A woman's understanding of her breast cancer risk is, therefore, potentially important in her choice of breast cancer screening options or risk-reduction strategies. The ability to clearly and accurately convey the estimate of breast cancer risk is a vital component of patient education that can enable a woman to make an informed decision. Previous studies have shown that women tend to overestimate their risk of breast cancer [2,3]. To decrease such misinterpretations of risk, it is imperative that women be presented information regarding their estimated risk of breast cancer in an understandable format tailored to their level of understanding.

In a report on risk communication, Lipkus and Hollands [4] showed that visual displays enhance the understanding of numerical risk. Furthermore, in a qualitative study using focus groups, women preferred a frequency format diagram to probability estimates for communicating risk estimates [5]. A review of the literature addressing the efficacy of breast cancer risk communication showed that, of several modalities used to communicate risk, no single modality was the most efficacious [6]. In a study assessing which formats are most accurately perceived by patients, Feldman-Stewart et al [7] reported that, for making a choice, systematic ovals, bars (horizontal or vertical), and numbers were equally well perceived, whereas for estimating magnitude of risk, numbers led to the most accurate estimates. Bogardus and colleagues [1] emphasized the importance of research for ascertaining the best techniques to communicate risks in the clinical setting. Although several studies have analyzed risk communication, few have been randomized trials [2], and none, to our knowledge, have been randomized trials comparing the efficacy of specific formats of communicating risk among women at high risk for breast cancer.

We conducted a prospective, randomized trial to compare communication of breast cancer risk using a bar graph (standard of care) versus the bar graph in addition to a frequency format diagram (using highlighted human figures) among women at increased risk of breast cancer. The aim of this study was to determine whether patient education regarding breast cancer risk using a bar graph alone or with the addition of a frequency format diagram improved the accuracy of risk perception and to assess women's preference for risk information provided as a bar graph versus in a frequency format.

## Methods

### Study population

In 2005, women aged 40 years or older presenting to the Breast Diagnostic Clinic at Mayo Clinic, Rochester, Minnesota, and considered to be at "increased breast cancer risk" (defined below) were eligible for the study. They were approached by the study coordinator to determine their interest in participation in the study and were provided detailed information regarding the study; interested persons provided informed consent. Women with prior breast cancer or lobular carcinoma in situ, receiving chemoprevention therapy (with tamoxifen, raloxifene, or in a chemoprevention trial), or with a history of prophylactic mastectomy were excluded. The study was approved by the Mayo Clinic Institutional Review Board.

### Definitions

#### Increased risk

Women were considered at "increased risk" 1) if the Gail model risk estimate was greater than 1.66% for development of invasive breast cancer over the next 5 years, 2) if a breast biopsy showed atypical hyperplasia, 3) if at least 1 first-degree relative had breast cancer, or 4) if more than 2 second-degree relatives had breast cancer or ovarian cancer [8,9].

#### Previsit questionnaire

All participants completed a previsit questionnaire immediately preceding the visit, providing information on educational status, Gail model risk factors, and current plans for screening or risk reduction. Participants' own perception of their breast cancer risk was assessed as a 5-year estimated risk of invasive breast cancer using numerical values (< 2%, 2%–9%, 10%–49%, 50%–74%, or ≥ 75%).

#### Randomization

**Figure 1:**
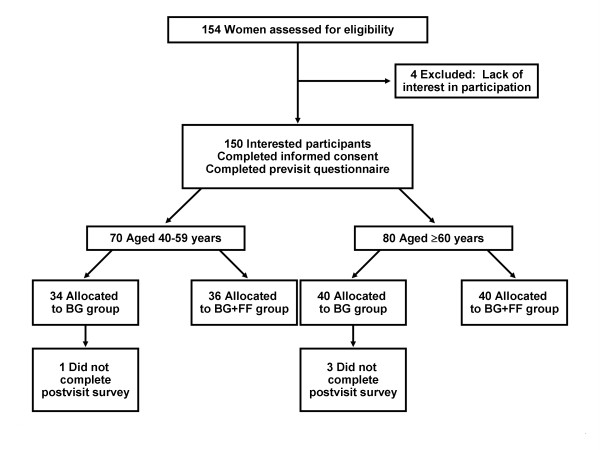
**Flow of the patients through the study.** BG group, received bar graph only; BG+FF group, received bar graph plus a frequency format diagram.

The standard of care in our practice has been to use a bar graph to communicate breast cancer risk estimates. Hence, all participants received a bar graph. Subjects were first stratified by age (40–59 years ["younger"] and ≥ 60 years ["older"]); within each age group, subjects then were randomly assigned to 1 of 2 groups: one to receive a bar graph alone (BG group) and the other to receive both a bar graph and a frequency format diagram (BG+FF group) (Figure 1). A stratified and dynamic randomization technique was used [10].

#### Bar graph and frequency format diagram

**Figure 2:**
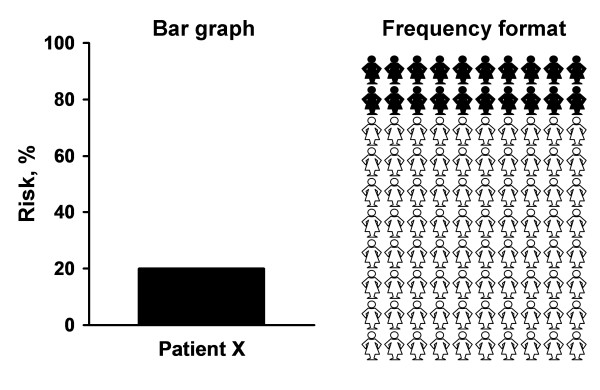
**Two methods of communicating risk.** The bar graph and frequency format diagram depict a 20% 5-year estimated Gail model risk of invasive breast cancer in a hypothetical patient X.

Each patient's 5-year risk estimate of invasive breast cancer was calculated using the Gail model [8]. Each patient in the BG group received a personalized bar graph (probability format) illustrating her 5-year risk estimate (Figure 2). Each participant assigned to the BG+FF group received a bar graph as noted above, as well as a frequency format diagram that pictorially showed consecutively highlighted human figures to indicate the 5-year risk of invasive breast cancer (Figure 2). A breast clinic medical provider discussed the patient's breast cancer risk estimate using the format or formats described above and addressed breast cancer risk-reduction strategies with the patient.

#### Postvisit questionnaire

Immediately after the visit with the breast clinic medical provider (including risk counseling), each participant completed a postvisit questionnaire before leaving the breast clinic. The questionnaire assessed the patient's breast cancer risk perception (5-year estimated risk of breast cancer using numerical values), preference for the bar graph or the frequency format diagram (for women in the BG+FF group), plans for future breast cancer screening options and risk-reduction strategies, and satisfaction with the information provided during the counseling visit. Our report focuses on risk perception and format preference only.

### Statistical analyses

The demographic characteristics of the study participants were calculated as frequencies (percentages) or mean ± SD and were compared between the 2 groups using the Pearson χ^2 ^test or 2-sample *t *test, respectively. "Accurate risk perception" was defined as the participant's perceived risk estimate category matching her actual Gail model risk estimate category. Intention-to-treat analysis was used such that if a participant did not complete the postvisit questionnaire, the postvisit risk score was assigned the same score as the previsit score. The accuracy of risk perception was compared between the 2 groups using the Pearson χ^2 ^test or logistic regression analysis to adjust for other factors. The difference in risk perception scores between the previsit and postvisit questionnaires was analyzed using the Wilcoxon rank sum test. The signed rank test was used to detect whether the risk score improved within each group. All *P *values less than .05 were considered statistically significant.

## Results

Of the 154 subjects who expressed an interest in the study, 150 provided informed consent and completed the previsit questionnaire; 74 patients (34 younger, 40 older) were randomly assigned to the BG group and 76 to the BG+FF group (36 younger, 40 older) (Figure 1). Four participants did not complete the postvisit questionnaire; all of them were in the BG group and were included in the study analyses (as detailed in the Methods).

Participants' demographic characteristics are shown in Table 1. Mean ± SD age of the study participants was 60.2 ± 10 years (range, 41–83 years). Most subjects were white (88%), had at least a high school education (97%), and had at least some college education (70%). No significant differences were observed between the 2 groups with regard to education, Gail model 5-year risk of invasive breast cancer, family history of breast cancer, number of prior breast biopsies, use of hormone replacement therapy, menopausal status, or previsit risk perception (Table 1).

**Table 1:** Demographics of the study population

**Variable**	BG group* (n = 74)	BG+FF group* (n = 76)	***P *value**^†^
**Mean ± SD Age, years**	60.9 ± 9.8	59.6 ± 10.2	.44^‡^

**Education**			.32

Elementary	4 (5%)	0 (0%)	

High school	21 (28%)	20 (26%)	

Some college	21 (28%)	25 (33%)	

College graduate	11 (15%)	14 (18%)	

Graduate or professional degree	17 (23%)	17 (22%)	

**Mean ± SD Gail risk score**	3.7 ± 2.1	3.2 ± 1.7	.12^‡^

**Gail risk score categories**			.51

< 2%	15 (20%)	11 (14%)	

2%–9%	57 (77%)	64 (84%)	

10%–49%	2 (3%)	1 (1%)	

≥ 50%	0 (0%)	0 (0%)	

**Family history**			.28

No	14 (19%)	20 (26%)	

Yes	60 (81%)	56 (74%)	

**Prior biopsy**			.24

None	36 (49%)	25 (33%)	

1	20 (27%)	25 (33%)	

2	12 (16%)	19 (25%)	

≥ 3	6 (8%)	7 (9%)	

**HRT use**			> .99

Never used	35 (47%)	36 (47%)	

Used	38 (51%)	39 (51%)	

Unknown	1 (1%)	1 (1%)	

**Menopause**			.14

No	13 (18%)	21 (28%)	

Yes	61 (82%)	55 (72%)	

HRT, hormone replacement therapy.

*Values are number of subjects (%) unless otherwise stated.

^†^Pearson χ^2 ^test.

^‡^Using 2-sample *t *test.

The Gail model 5-year risk of invasive breast cancer was between 2% and 9% for 81% of the overall cohort; 17% had a risk score of < 2%, and 2% of the sample had a score of 10% to 49% (Table 1). On the previsit questionnaire, 72% of the overall cohort overestimated their risk of breast cancer.

**Figure 3:**
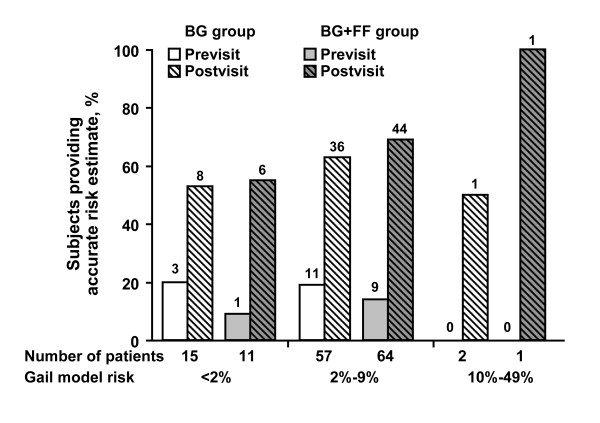
**Proportion of subjects providing accurate previsit and postvisit estimates of breast cancer risk.** Subjects are stratified by group (BG or BG+FF) and their actual Gail model level of risk. Bars reflect the percentage of accurate previsit and postvisit estimates of risk based on the number of women accurately estimating that level of risk (numbers above bars) and the number of women in each risk category (numbers below bars).

In the overall assessment of risk perception, accurate risk perception was noted for 14 women (4 younger, 10 older; 19%) in the BG group before the visit and improved to 45 women (21 younger, 24 older; 61%) after the visit, whereas in the BG+FF group, the accuracy of risk perception improved from 10 women (6 younger, 4 older; 13%) before the visit to 51 women (26 younger, 25 older; 67%) after the visit. The previsit to postvisit changes in accurate risk perception are detailed in Figure 3. Of the 15 women in the BG group with risk score < 2%, 3 (20%) had accurate risk perception before the visit; this increased to 8 patients (53%) after the visit. For the 11 women in the BG+FF group with risk score < 2%, accurate risk perception increased from 1 patient (9%) previsit to 6 patients (55%) postvisit. The overall difference in level of improvement between the BG and BG+FF groups was not significant (*P *= .10 by the Pearson χ^2 ^test and *P *= .29 by logistic regression when previsit score and age were in the model); age also did not affect the scores (*P *= .57).

The results of the previsit and postvisit questionnaires, depicting patients' self-reported estimates of their risk compared with the actual Gail risk model results, are shown in Table 2. Although 121 (81%) of the overall group had a Gail risk score of 2% to 9%, only 15 women (20%) in the BG group and 13 women (17%) in the BG+FF group estimated this risk on the previsit questionnaire, with 11 and 9 women, respectively, giving accurate responses (Figure 3). However, on the postvisit questionnaire, 40 women (54%) in the BG group and 47 (62%) in the BG+FF group estimated this level of risk, which was an accurate risk estimation for 36 and 44 women, respectively. This increase constituted an improvement in risk estimation for both groups, but the difference between groups was not significant (*P *= .17).

**Table 2:** Patient-reported risk perception (previsit and postvisit) and actual Gail model risk score

	BG group (n = 74) *			BG+FF group (n = 76) *		
**Gail model risk category**	**Actual**	**Previsit**	**Postvisit**	**Actual**	**Previsit**	**Postvisit**

**< 2%**	15 (20%)	12 (16%)	10 (14%)	11 (14%)	12 (16%)	12 (16%)

**2%–9%**	57 (77%)	15 (20%)	40 (54%)	64 (84%)	13 (17%)	47 (62%)

**10%–49%**	2 (3%)	26 (35%)	10 (14%)	1 (1%)	34 (45%)	15 (20%)

**50%–74%**	0	14 (19%)	10 (14%)	0	16 (21%)	2 (3%)

**≥ 75%**	0	7 (9%)	4 (5%)	0	1 (1%)	0

*Values are number of subjects (%) in each category of breast cancer risk.

Although none of the women in the study had Gail model risk estimates of 50% or greater, 21 women (28%) in the BG group and 17 (22%) in the BG+FF group reported such high risk on the previsit questionnaire (Table 2). Of interest, after risk education, 14 women (19%) in the BG group still reported risk of 50% or higher, whereas only 2 women (3%) in the BG+FF group reported this high level of risk (*P *= .004). The difference between previsit and postvisit perception of high risk was greater in the BG+FF group (88% decrease) than in the BG group (33% decrease) (*P *< .001).

In response to the question assessing preference for presentation of risk information, asked only of women who saw both formats (BG+FF group), 34% (n = 26) "preferred frequency format," 15% (n = 11) "preferred bar graph," 43% (n = 33) felt that "both were just as good," and 8% (n = 6) had "no preference/don't know."

## Discussion

Understanding breast cancer risk is a vital component of informed and participatory decision making [1,11]. Accurate understanding of risk could potentially affect adherence to recommendations for breast cancer screening, evaluations for breast cancer diagnosis, selection of treatment options for breast cancer, and strategies to decrease the risk of breast cancer in women at high risk. Breast cancer risk estimates must, therefore, be communicated to women in a manner that is easily understandable. This study suggests that the use of a frequency format diagram in addition to a bar graph to communicate breast cancer risk can provide added benefit, especially for women who inaccurately perceive very high risk.

Combining visual displays with numerical and written information can be effective in communicating risk information [12]. In our study, accurate risk perception for the overall study population was reported by 16% of all participants before the visit, whereas after risk communication using pictorial depiction of risk and verbal communication, 64% of participants reported accurate risk. Although risk perception improved after the visit with the medical provider, there is still need for further improvement, and these findings support the need for continued research to enhance the risk communication process. These findings also are comparable to previous reports of women receiving breast cancer risk information that showed short-term improvement in the accuracy of risk perception after an educational visit [13,14]. Lipkus et al [14] also reported that presenting risk as a point estimate or as a range of risks decreased women's estimates of their risks, but the women continued to overestimate their risk relative to their Gail model risk scores.

In our study, no significant difference was noted in the accuracy of risk perception between the BG group and the BG+FF group (*P *= .10). Among women in the 2% to 9% risk category, discussion using either the bar graph alone or the bar graph plus the frequency format diagram improved the accuracy of risk perception, but the difference between the groups was not statistically significant. In contrast, among women who inaccurately perceived very high risk (≥ 50%), the BG+FF group had significantly improved accuracy of risk perception compared with the BG group (*P *= .004). In a previous report, Gigerenzer [15] stated that Bayesian computations are easier when the information is in a frequency format rather than in a probability format. Timmermans et al [11] used different formats for communicating surgical risk to investigate the effect of format on participants' interpretation of risk and choice of treatment; they reported that vertical bars were the most difficult to comprehend, whereas information in the form of icons was most helpful for making a decision. A frequency format diagram in addition to a bar graph for depiction of a risk estimate may, therefore, improve the accuracy of risk perception by highlighting or clarifying low-risk status, especially among women inaccurately perceiving high risk.

In a study of the effect of graphic format on perception of risk magnitude, Schapira et al [16] reported that numerical lifetime risk was perceived to be of lower magnitude when the subject was presented with a bar graph compared with a pictorial display. They suggested that an affective (emotional) response, resulting from identification with the numerator of the risk estimate, may explain their findings. Their study also reported that risk was perceived to be of lower magnitude when symbols in the frequency format diagram were highlighted consecutively rather than randomly. In the current study, we highlighted the human figures consecutively and found an enhanced understanding of lower risk in the BG+FF group. An interesting area for future research would be to assess whether the size and characteristics of the display (eg, consecutive highlighting), rather than the magnitude of risk itself, affect risk perception.

In a study of risk communication among medical inpatients aged 75 years and older, Fuller et al [17] found that pictorial representation of probability with human figures was better understood than simple verbal statements. A subsequent report studying a younger population of inpatients showed that patients of all ages have the potential to misinterpret numerical probability information and that pictorial description of risk was well understood by all patients; the authors advocated the use of such tools in clinical practice to communicate risk [18]. In the current report, we found that age had no effect on the accuracy of risk perception.

Previous studies of breast cancer risk perception found that women tend to overestimate their risk [2,3]. In this study, 72% of women overestimated their risk on the previsit questionnaire. Even after intervention, 31% still overestimated their risk, which was not influenced by age, education, family history, or use of hormone replacement therapy. This finding highlights the need to objectively evaluate additional strategies to enhance the understanding of breast cancer risk.

The question of preference for format was addressed in the BG+FF group. One-third of these participants preferred the frequency format diagram, and 43% believed that it was just as good as the bar graph. Gigerenzer and Edwards [19] reported that frequency statements foster insight and decrease confusion when discussing single-event probabilities. In another study, Schapira et al [16] reported that a pictorial display was preferred to a bar graph for presentation of single risks. Participants in that study preferred consecutive rather than random highlighting of the symbols in the pictorial display. Therefore, it may be reasonable to incorporate the frequency format diagram into clinical practice for breast cancer risk communication.

The strength of this study lies in the prospective, randomized study design, which enabled assessment of different formats for risk communication between 2 comparable groups of high-risk women. Women presenting to the breast clinic were participants in the study, and these results can be generalized to similar groups of high-risk women. We acknowledge, however, that this study has some limitations. The participant population was mainly white and had at least a high school education, which suggests that results may not be generalizable to all populations. In addition, numeracy could have affected understanding but was not addressed in this study and may have provided further insight into the study findings. Furthermore, we could not assess or control for the providers' oral explanations of breast cancer risk that could potentially have contributed to allocation bias, but providers were advised to use only the format or formats of communicating risk assigned to them for this study. Additional research is needed to assess retention of information over time because this study only assessed risk perception immediately after the visit.

## Conclusion

In summary, this study showed that breast cancer risk communication using a bar graph along with a frequency format diagram can improve the accuracy of risk perception, especially among women perceiving inaccurately high risk. Many women may prefer the frequency format, suggesting that incorporating both of these formats into clinical practice to communicate breast cancer risk is reasonable. Future studies to include larger populations of women with varying risk and educational status, and longer-term follow-up of the participants to assess sustained understanding of risk, are indicated in our efforts to enhance risk communication.

## Competing interests

The authors declare that they have no competing interests.

## Authors' contributions

KG, AKG, BJC, SP, CIW, REJ, DW-R, MKB, and SSC were involved in study conception and design. Data acquisition was performed by KG, BJC, SP, CIW, LN, NPS, REJ, DW-R, and MKB. SSC and KG performed the statistical analyses. KG drafted the manuscript, and all authors read and approved the final manuscript.

## Pre-publication history

The pre-publication history for this paper can be accessed here:


